# Perceived stress levels of pet and non-pet owners in Cebu, Philippines

**DOI:** 10.5455/javar.2026.m1027

**Published:** 2026-03-16

**Authors:** Adrian Patalinghug Ybañez, Michelle Plaza Trangia, Grahambell Bacalla Sabanate, Marry Rose Celeste Estrera, Maria Maica Sofia Estenzo Teñoso, Ron Adriel Lylm Arriesgado Lauron, Shem Lim Alberio, Ruby Herseniada Destajo, Serafin Limosnero Garciano, Rochelle Haidee Palermo Daclan Ybañez, Marvin Ardeza Villanueva

**Affiliations:** 1 Institute for Molecular Genetics, Parasitology and Vector-Borne Diseases, Cebu Technological University, Cor. M.J. Cuenco Ave. and R. Palma St., Cebu City 6000, Philippines; 2 College of Veterinary Medicine, Barili Campus, Cebu Technological University, Cagay, Barili, Cebu 6036, Philippines; 3 Department of Psychology and Social Sciences, College of Arts and Sciences, Cebu Technological University, Cor. M.J. Cuenco Ave. and R. Palma St., Cebu City 6000, Philippines; 4 Biosafety and Environment Section, Research and Development Division, Philippine Carabao Center, National Headquarters and Gene Pool, Science City of Muñoz, Nueva Ecija 3119, Philippines

**Keywords:** Cebu, Philippines, pet ownership, pet-related stressors, stress level

## Abstract

**Objectives:** Owning pets has been shown to reduce stress and improve the well-being of their owners in other countries. The present study aimed to document the perceived stress levels among pet and non-pet owners in the Philippines. In this country, the literature about this is still limited.

**Materials and Methods:** Using a descriptive-analytical design, 417 respondents (286 pet owners and 131 non-pet owners) were asked about their stress status, personal stressors, coping mechanisms (including exercise or social support), impacts of physical activity levels, well-being, support systems, and the effect of having a pet on stress.

**Results:** Pet ownership was more prevalent among females and younger individuals, particularly those aged 18–25. A statistically significant association was observed between pet ownership and being single (*p* = 0.019). However, this does not necessarily imply that single people were more likely to have pets for companionship, as the majority of respondents were single. The study also revealed that pet owners experience lower stress than non-pet owners. Significant differences were observed in coping mechanisms (*p* < 0.01), the impact of pet ownership on stress (*p* < 0.01), and overall stress levels (*p* < 0.01), highlighting the vital role pets play in emotional support and stress management. Pet ownership was also significantly linked to physical activity levels (*p* = 0.043) and personal stressors (*p* = 0.003). Despite these benefits, some pet owners reported challenges, including financial and time constraints.

**Conclusions:** The findings indicate that while owning a pet comes with specific responsibilities, it is a valuable way to cope with stress and supports mental health and well-being.

## 1. Introduction

Stress is a significant aspect of human life, affecting physical, mental, and emotional health. Various strategies have been proposed to control stress, including social networks, lifestyle changes, and pet interactions [1]. The use of pets has been shown to help individuals manage stress levels and provide emotional support. In adolescents, prolonged interaction with pets increases the “relating to others” domain, as pets provide social support and opportunities to build new social connections [2]. This benefit was particularly evident during the COVID-19 pandemic, when pet ownership significantly influenced individuals’ psychological well-being worldwide [3].

Owning pets is linked to favorable physiological effects, including reduced blood pressure, reduced anxiety, and enhanced mental health [4]. It has been shown to affect an individual’s psychosocial functioning and to reduce cortisol production. It produces dual effects by reducing social isolation and fostering stronger social bonds, thereby augmenting personal happiness [5]. These findings highlight the benefits of pet ownership, especially as the number of households with pets continues to rise owing to urbanization, social isolation, and increased recognition of these benefits [6].

There is a need for further research to fully understand the relationship between pet ownership and stress reduction. These findings could provide policymakers and health professionals with valuable insights for developing more effective strategies to combat stress, particularly in areas undergoing rapid urbanization. If pet ownership reduces stress, animals could be integrated into programs to improve mental well-being. As mental health issues continue to rise, non-drug interventions, such as animal-assisted therapy and interactions with pets, could offer alternative methods for managing stress. Hence, further understanding the role of pet ownership and its potential use for effective stress management strategies is advantageous [7].

While pet ownership has been associated with reduced stress and improved well-being, its specific impacts in the Philippine context remain primarily unexplored [8]. The COVID-19 pandemic has caused significant psychological distress among Filipinos [9], and human-animal interactions have been shown to have buffering effects on stress and quality of life during the crisis [10]. In the post-pandemic period, the potential benefits of pet ownership in the Philippines can be explored amid significant changes in social and environmental dynamics. Better understanding could lead to the development of effective stress-reduction techniques tailored for pet and non-pet owners in the Philippines, with potential applicability in other countries [11].

Pet ownership may seem advantageous, but several factors influence it. Notable differences in stress levels persist between pet owners and non-owners, especially among people with higher socioeconomic status [12, 13]. The potential advantages of having a pet may not be equally experienced across social groups [14]. Moreover, region-specific studies on pet ownership are lacking, limiting understanding of its role in stress management in contexts such as the Philippines. This limitation may restrict the applicability of the results to the Filipino community and its distinctive relationship with pet ownership and stress management [15].

Cebu warrants specific attention due to its rapid urbanization and diverse socioeconomic landscape. The combination of urban and rural lifestyles creates a unique environment where traditional cultural norms intersect with modern stressors, generating distinct stress patterns. Economic and cultural factors shape attitudes toward pet companionship in Cebu in ways that broader studies cannot capture [16]. Additionally, Cebu’s population experienced notable psychological impacts during the COVID-19 pandemic, providing a timely opportunity to explore how human-animal interactions buffer stress locally [17]. Understanding the role of pet ownership in stress reduction in Cebu is crucial for designing culturally appropriate interventions. Such insights can guide policymakers and health professionals in adopting non-pharmacological strategies such as animal-assisted therapy [18].

Despite existing studies suggesting potential mental health advantages of pet ownership, there is insufficient empirical evidence to determine whether pet owners experience lower stress levels than non-owners and how demographic factors might influence these differences in Cebu, Philippines. Pet owners may experience multiple difficulties, which include pet caretaking obligations along with high veterinary costs and intense psychological stress from their animals’ medical conditions or death/loss. Thus, the present study aimed to compare stress levels among pet owners and non-pet owners in Cebu, Philippines, and to offer a better understanding of the potential mental health benefits of pet companionship within the local cultural context.

## 2. Materials and Methods

### 2.1. Ethical approval

All participants provided informed consent and received assigned identifiers to protect their anonymity and privacy. This reduced the likelihood of unintended identification. All procedures complied with the ethical standards of the Philippine Health Research Ethics Board and were in accordance with the Philippine National Health Research System Act of 2013 (Republic Act No. 10532).

### 2.2. Data protection

Survey participants received absolute privacy protection, as their personal data remained confidential. Their personal information was withdrawn from raw datasets. Only research team members have exclusive access to survey responses to preserve confidentiality, in accordance with confidentiality agreements. The obtained data is exclusively used for the research. All participants provided informed consent and received assigned identifiers to protect their anonymity and privacy. This reduced the likelihood of unintended identification. All procedures complied with the ethical standards of the Philippine Health Research Ethics Board and were in accordance with the Philippine National Health Research System Act of 2013 (Republic Act No. 10532) [19]. All participants had the opportunity to view their information before final submission and to withdraw any study-related details at any time.

### 2.3. Locale of the study

The research area selected was Cebu, Philippines, due to its multicultural environment, active economy, and substantial pet population. As the country’s second-largest city and most prosperous province, Cebu faces distinct urban problems alongside financial difficulties, social stresses, and limited access to mental health services. Stress reduction through pet ownership can be significant in Cebu, considering its situation. This study is of great importance because Cebu’s rapid growth and rising pet ownership rates create ideal conditions for research on human-animal interactions and urban Filipino mental health.

### 2.4. Participants

A total of 417 respondents (286 pet owners and 131 non-pet owners) participated in the study. Respondents were purposively selected. The inclusion criteria included age (18 years and above) and location (residing in Cebu for at least six months). To qualify for the study, pet owners must currently own at least one pet (e.g., dogs, cats, or birds) and have owned a pet for at least two weeks. Non-pet owners are individuals who do not currently own pets.

### 2.5. Instrument

The questionnaire included separate sections to collect demographic and stress-assessment information from survey participants. Demographic data comprised four components: names, addresses, ages, sex preferences, and civil status, together with pet owner data, animal information, and ownership schedules. The main section achieved construct validity by presenting five statements for each key area that assessed personal stressors, coping mechanisms, physical activity levels, well-being evaluations, support systems, and perceived stress impacts from pet ownership (30 statements in total). The constructs were based on the concept of measuring stress through personal stressors and coping strategies, aligning with the transactional model of stress by Lazarus and Folkman [20]. Furthermore, the use of Likert-scale statements to measure agreement with stress-related attitudes and behaviors reflects common standardized methods seen in psychological assessments [21].

The emphasis on physical activity’s role in stress management aligns with evidence linking exercise to stress reduction via physiological and psychological pathways [22]. This supports the inclusion of items in the questionnaire that assess how physical activity influences stress levels. Additionally, social support, as a key protective factor against stress, is well documented, with Cohen and Wills [23] highlighting the buffering effects of emotional and instrumental support from family and friends, which justify the questionnaire’s comprehensive approach to support systems. Notably, the inclusion of pet ownership-related items is backed by accumulating research demonstrating the stress-relieving benefits of companion animals. Studies indicate that interacting with pets can improve mood, lower blood pressure, and provide emotional comfort, all of which contribute to psychological well-being [24].

Two open-ended questions were included in the questionnaire to understand how pet owners experience the advantages and difficulties of pet ownership for stress management. The main section showed a high internal reliability coefficient (Cronbach’s alpha = 0.85), indicating that the items reliably measured stress-related constructs. Additionally, inter-observer reliability was ensured by standardizing behavioral categories, thereby maintaining consistency in data analysis across evaluators.

The Cronbach’s alpha analysis of the instrument indicates that the values fall within the range generally considered acceptable for psychological scales (alphas of 0.7 or higher suggest that the items consistently measure the same underlying construct). The personal stressors scale, with an alpha of 0.78, demonstrates good internal consistency, indicating that the questions reliably capture respondents’ personal stress. Similarly, the coping mechanisms and well-being scales, with alphas above 0.8, suggest that these items are closely related and reliable in assessing how individuals manage stress and perceive their overall well-being. The support system scale’s alpha is near 0.79, suggesting that items on social connections and emotional support are cohesive, which is essential when evaluating social factors that buffer stress. Notably, the scale measuring the impact of pet ownership on stress shows the highest alpha at 0.84, indicating strong internal reliability in examining how pet ownership relates to stress relief symptoms.

### 2.6. Procedure

Potential participants were contacted using purposive and snowball sampling techniques. After confirmation of participation, an online survey utilizing Google Forms was sent. Upon reaching the quota, the online data were imported into Microsoft Excel and further coded. Processed data were imported into the Statistical Package for the Social Sciences (SPSS) software for further analysis using descriptive statistics and the Chi-square test, where applicable. Variables with *p* values equal to or less than 0.05 were considered significant.

### 2.7. Data analysis

Descriptive statistics was used to summarize the characteristics and stress levels within the two respondent groups. To explore differences between pet owners and non-pet owners, the Chi-square test was used. The standard significance threshold was set at *p* = 0.05. All analyses used the IBM Statistical Package for the Social Sciences (SPSS).

## 3. Results and Discussion

### 3.1. Profile of pet owners and non-pet owners

Overall, most respondents were female (70%), between 18 and 25 years of age (85.4%), and single (91.4%) (Table 1). This trend showed a higher proportion among pet owners than among non-pet owners, suggesting that younger, female, and single individuals are more likely to own pets. The research found that dog ownership is associated with better mental health among owners, especially women. The test participants reported reduced stress and anxiety, along with improved mood and higher motivation. Dog owners felt socially connected and deeply emotionally supported through their relationships with their pets. Females are more inclined toward nurturing roles or companionship-seeking behavior that aligns with pet ownership [25]. Additionally, societal norms often associate caregiving and emotional connection with females, potentially driving their interest in pet ownership. Marketing and media also frequently target women as primary caregivers for pets, reinforcing this pattern [26].

**Table 1.** Demographic profile of pet owners and non-pet owners in Cebu, Philippines (*n* = 417).

Parameter	Pet owner		Non-pet owner		Total	
	Freq	%	Freq	%	*n*	%
Biological Sex at Birth						
Female	203	48.7	89	21.3	292	70.0
Male	83	19.9	42	10.1	125	30.0
Total	286	68.6	131	31.4	417	100.0
Age Category						
18–25	250	60.0	106	25.4	356	85.4
26–35	27	6.5	17	4.1	44	10.6
above 35	9	2.2	8	1.9	17	4.1
Total	286	68.6	131	31.4	417	100.0
Marital Status						
Single	267	64.0	114	27.3	381	91.4
Married	16	3.8	10	2.4	26	6.2
Live-in	2	0.5	7	1.7	9	2.2
Separated	1	0.2	0	0.0	1	0.2
Total	286	68.6	131	31.4	417	100.0
Type of Animal Owned*						
None	–	–	131	31.4	131	31.4
Dog	200	48.0	–	–	195	46.8
Cat	78	18.7	–	–	74	17.7
Bird	5	1.2	–	–	3	0.7
Livestock species	4	1.0	–	–	2	0.5
Aquatic Animals	7	1.7	–	–	5	1.2
Rabbit	1	0.2	–	–	1	0.2
Hamster	1	0.2	–	–	1	0.2
Total	286	68.6	131	31.4	417	100.0
Years of pet ownership						
0	–	–	131	31.4	131	31.4
Less than 1	4	1.0	–	–	4	1.0
1–3	85	20.4	–	–	86	20.6
4–6	85	20.4	–	–	87	20.9
7–9	31	7.4	–	–	34	8.2
10 and above	81	19.4	–	–	85	20.4

*Multiple answers.

On the other hand, the above-mentioned observation could also be attributed to their relatively flexible schedules, increased social media influence promoting pet ownership, and the growing trend of finding companionship in animals during this phase of life [4]. In older age brackets, pet ownership may decline significantly due to considerations related to life stages or responsibilities that limit their capacity to care for pets, including raising children [27]. Pet ownership was associated with household size, country of residence, economic status, and marital status. Single individuals may have greater freedom to adopt pets, as they are not constrained by shared decision-making or the responsibilities of managing a household with a partner or children [28].

Dogs (69.9%) were the most common pets among pet owner respondents, followed by cats (27.3%). Some owners mentioned owning multiple species. Dogs may have been preferred for their protective, familial, and companionship roles [29]. Cats are also popular because they are independent, making them suitable for people with hectic schedules. Compared to dogs and cats, other species may be less familiar or accessible, which could explain their low numbers [30].

Pet ownership duration varied widely, with most pet owners reporting one to six years of ownership. Durations of less than a year were uncommon, indicating that pet owners in the study tended to maintain longer-term commitments to their pets. This trend highlights owners’ sense of responsibility and attachment, reflecting the emotional bonds formed over time [31]. Long years of pet ownership may point to a tradition or lifestyle where pets are integral to family life, passed down across generations, or continuously maintained as a source of companionship and well-being [32].

### 3.2. Perceived stress levels of participants on different key stress areas

Results of personal stressors revealed that most pet owners and non-pet owners experienced moderate stress levels (70% and 60.7%, respectively). However, more individuals without pets reported high stress (38.6%) compared to those with pets (29.3%). This difference might be attributed to the potential stress-alleviating effects of pet companionship, including emotional support and a sense of purpose that can divert attention from personal challenges. This emphasis on the potential stress-alleviating effects of pet companionship can make the audience feel hopeful and optimistic [4, 33].

In contrast, those without pets may lack this outlet, potentially leading to higher stress levels. Regarding coping mechanisms, most respondents fell into the medium-stress category (62.8% among pet owners and 69.3% among non-pet owners). Interestingly, pet owners were more likely to be in the high-stress category (36.9%) than non-pet owners (30%). This finding may suggest that pet owners face unique challenges related to their animals, such as managing pet-related responsibilities or expenses, which could elevate their stress when coping. This emphasis on the unique challenges faced by pet owners can make the audience feel respectful and considerate [34]. Nevertheless, their access to stress-reducing activities, such as interacting with pets, is anxiety-relieving [28].

Regarding physical activities, both groups predominantly exhibited medium stress levels, with non-pet owners showing slightly higher levels (72.9%) than pet owners (68.6%). A similar percentage of individuals with pets (11.7%) and those without (10%) reported low stress levels. Incidental physical activity, such as walking or playing with pets, may help pet owners mitigate the adverse effects of stress [33, 35]. Conversely, individuals without pets may have fewer opportunities for regular exercise, potentially leading to slightly higher medium stress levels [36].

A larger proportion of non-pet owners reported high levels of personal stressors, well-being, and support systems than pet owners (Table 3). Pet ownership can positively impact mental well-being, possibly through companionship and emotional support [37]. Furthermore, it may complement human social support networks. A study by Marí-Klose et al. [38] underscores the importance of considering dog ownership and family involvement in care strategies to mitigate loneliness in age-dependent dependents with mobility impairments.

Pet owners seem to need more support with coping mechanisms and physical activity, as many reported high stress levels. While both groups struggle with stress management, pet owners might rely on their animals as a coping strategy and for additional physical activities [39]. Participating in physical activities, such as walking or playing with pets, enhances stress management.

Most pet owners (79.4%) reported medium stress levels related to pet ownership, with only 2% experiencing high stress. In comparison, 15.7% of non-pet owners reported high-stress levels in this category. The presence of pets may offer emotional benefits that counterbalance potential pet-related challenges, such as financial costs or caretaking responsibilities, fostering a sense of empathy and understanding among the audience. Having pets has a beneficial effect on mental health. Participants reported feeling less stress and anxiety, an uplift in spirits, and increased motivation [25].

A significant association was observed between pet ownership and civil status (*p* = 0.019) (Table 3), suggesting that single individuals may be more likely to own pets than married couples, as the latter may have other priorities than owning pets or see it as an added responsibility. This suggests that the motivation to own pets may differ by relationship status, with single individuals possibly seeking emotional support and companionship from pets [14]. Married couples may also distribute pet-related responsibilities if they own a pet [4].

On the other hand, the overall stress levels of pet owners and non-pet owners were significantly different (*p* < 0.01) (Table 3), suggesting that pet ownership may influence stress experiences. Additionally, pet ownership was significantly associated with physical activity levels (*p* = 0.043) and personal stressors (*p* = 0.003). These findings highlight the complex interplay between pet ownership and stress-related factors [29].

### 3.3. Pet owners’ perception of the pet’s role in stress management

Several pet owner respondents (51.5%) reported that their animals provided emotional support and companionship (Table 2), underscoring pets’ crucial function in alleviating stress and enhancing psychological health. This finding is consistent with earlier research suggesting that pet companionship can enhance feelings of security, mitigate loneliness, and offer solace during challenging times [40]. Moreover, 24% of participants appreciated the unconditional affection they received from their pets, reinforcing the idea that animals can be a steady source of positive emotional support [41]. Furthermore, 6% of pet owners recognized their animals’ role in promoting physical exercise, such as walking or playtime, contributing to bodily and mental wellness. Pet-related activities may be a coping strategy that lowers stress levels and encourages an active lifestyle [42].

**Table 2.** Responses towards the stress levels on specific constructs of the key stress areas among pet owners and non-pet owners (*n* = 417).

Statements	Type of ownership	Strongly disagree		Disagree		Agree		Strongly agree		Total	
		Freq	%	Freq	%	Freq	%	Freq	%	*n*	%
1. I find it difficult to manage stress in dealing with personal issues.	Pet owner	27	6.5	85	20.4	132	31.7	42	10.1	286	68.6
	Non-pet owner	10	2.4	42	10.1	56	13.4	23	5.5	131	31.4
	Total	37	8.9	127	30.5	188	45.1	65	15.6	417	100.0
2. I am easily affected by the financial concerns, which significantly heighten my overall stress.	Pet owner	31	7.4	75	18.0	126	30.2	54	12.9	286	68.6
	Non-pet owner	4	1.0	40	9.6	61	14.6	26	6.2	131	31.4
	Total	35	8.4	115	27.6	187	44.8	80	19.2	417	100.0
3. I frequently procrastinate, which significantly contributes to my stress levels.	Pet owner	11	2.6	74	17.7	152	36.5	49	11.8	286	68.6
	Non-pet owner	8	1.9	26	6.2	75	18.0	22	5.3	131	31.4
	Total	19	4.6	100	24.0	227	54.4	71	17.0	417	100.0
4. I have good control over personal factors, which lessens my stress.	Pet owner	18	4.3	55	13.2	124	29.7	89	21.3	286	68.6
	Non-pet owner	7	1.7	42	10.1	52	12.5	30	7.2	131	31.4
	Total	25	6.0	97	23.3	176	42.2	119	28.5	417	100.0
5. I have a strong sense of support from friends and family in managing my personal stress.	Pet owner	44	10.6	87	20.9	90	21.6	65	15.6	286	68.6
	Non-pet owner	11	2.6	44	10.6	38	9.1	38	9.1	131	31.4
	Total	55	13.2	131	31.4	128	30.7	103	24.7	417	100.0
6. I am NOT comfortable seeking help or counseling for stress-related issues.	Pet owner	73	17.5	107	25.7	68	16.3	38	9.1	286	68.6
	Non-pet owner	31	7.4	49	11.8	33	7.9	18	4.3	131	31.4
	Total	104	24.9	156	37.4	101	24.2	56	13.4	417	100.0
7. I doubt the effectiveness of relaxation techniques (e.g., deep breathing, meditation) to manage stress.	Pet owner	43	10.3	79	18.9	119	28.5	45	10.8	286	68.6
	Non-pet owner	28	6.7	48	11.5	37	8.9	18	4.3	131	31.4
	Total	71	17.0	127	30.5	156	37.4	63	15.1	417	100.0
8. Engaging in social events does little to alleviate my stress.	Pet owner	30	7.2	52	12.5	118	28.3	86	20.6	286	68.6
	Non-pet owner	11	2.6	29	7.0	49	11.8	42	10.1	131	31.4
	Total	41	9.8	81	19.4	167	40.0	128	30.7	417	100.0
9. I engage in relaxation techniques (e.g., deep breathing, meditation) to manage personal stress.	Pet owner	22	5.3	79	18.9	132	31.7	53	12.7	286	68.6
	Non-pet owner	3	0.7	35	8.4	58	13.9	35	8.4	131	31.4
	Total	25	6.0	114	27.3	190	45.6	88	21.1	417	100.0
10. I perceive social events as totally effective in alleviating my personal stress.	Pet owner	82	19.7	113	27.1	70	16.8	21	5.0	286	68.6
	Non-pet owner	27	6.5	47	11.3	41	9.8	16	3.8	131	31.4
	Total	109	26.1	160	38.4	111	26.6	37	8.9	417	100.0
11. My engagement in any physical activity increases my stress levels rather than reducing them.	Pet owner	94	22.5	112	26.9	62	14.9	18	4.3	286	68.6
	Non-pet owner	39	9.4	60	14.4	24	5.8	8	1.9	131	31.4
	Total	133	31.9	172	41.2	86	20.6	26	6.2	417	100.0
12. I find it difficult to relax and unwind after participating in physical activity.	Pet owner	99	23.7	113	27.1	57	13.7	17	4.1	286	68.6
	Non-pet owner	39	9.4	63	15.1	22	5.3	7	1.7	131	31.4
	Total	138	33.1	176	42.2	79	18.9	24	5.8	417	100.0
13. I find physical activity does NOT effectively help me cope in managing my stress.	Pet owner	121	29.0	102	24.5	51	12.2	12	2.9	286	68.6
	Non-pet owner	54	12.9	51	12.2	21	5.0	5	1.2	131	31.4
	Total	175	42.0	153	36.7	72	17.3	17	4.1	417	100.0
14. I find it easy to manage my stress during physical activity.	Pet owner	16	3.8	63	15.1	131	31.4	76	18.2	286	68.6
	Non-pet owner	4	1.0	27	6.5	65	15.6	35	8.4	131	31.4
	Total	20	4.8	90	21.6	196	47.0	111	26.6	417	100.0
15. I believe that increasing my physical activity levels has reduced my stress levels.	Pet owner	12	2.9	39	9.4	144	34.5	91	21.8	286	68.6
	Non-pet owner	4	1.0	22	5.3	57	13.7	48	11.5	131	31.4
	Total	16	3.8	61	14.6	201	48.2	139	33.3	417	100.0
16. I rarely experience physical symptoms of stress in my personal life (e.g., headaches, insomnia).	Pet owner	56	13.4	105	25.2	94	22.5	31	7.4	286	68.6
	Non-pet owner	23	5.5	50	12.0	41	9.8	17	4.1	131	31.4
	Total	79	18.9	155	37.2	135	32.4	48	11.5	417	100.0
17. My personal life is NOT affected by my overall stress levels.	Pet owner	85	20.4	114	27.3	75	18.0	12	2.9	286	68.6
	Non-pet owner	32	7.7	68	16.3	25	6.0	6	1.4	131	31.4
	Total	117	28.1	182	43.6	100	24.0	18	4.3	417	100.0
18. I rarely feel a sense of despair regarding my ability to effectively manage personal stress.	Pet owner	22	5.3	124	29.7	124	29.7	16	3.8	286	68.6
	Non-pet owner	8	1.9	67	16.1	53	12.7	3	0.7	131	31.4
	Total	30	7.2	191	45.8	177	42.4	19	4.6	417	100.0
19. I doubt that changes in my lifestyle or habits could significantly reduce my stress levels.	Pet owner	49	11.8	97	23.3	105	25.2	35	8.4	286	68.6
	Non-pet owner	15	3.6	52	12.5	40	9.6	24	5.8	131	31.4
	Total	64	15.3	149	35.7	145	34.8	59	14.1	417	100.0
20. I rarely engage in activities that bring me joy or relaxation due to personal stress.	Pet owner	52	12.5	105	25.2	89	21.3	40	9.6	286	68.6
	Non-pet owner	24	5.8	44	10.6	52	12.5	11	2.6	131	31.4
	Total	76	18.2	149	35.7	141	33.8	51	12.2	417	100.0
21. I feel connected and have a strong support system in dealing with challenges.	Pet owner	8	1.9	66	15.8	133	31.9	79	18.9	286	68.6
	Non-pet owner	4	1.0	37	8.9	62	14.9	28	6.7	131	31.4
	Total	12	2.9	103	24.7	195	46.8	107	25.7	417	100.0
22. I am satisfied with the emotional support I receive from friends and family.	Pet owner	11	2.6	49	11.8	123	29.5	103	24.7	286	68.6
	Non-pet owner	9	2.2	24	5.8	56	13.4	42	10.1	131	31.4
	Total	20	4.8	73	17.5	179	42.9	145	34.8	417	100.0
23. My conflict in personal relationships is NOT significantly contributing to my overall stress.	Pet owner	42	10.1	131	31.4	92	22.1	21	5.0	286	68.6
	Non-pet owner	23	5.5	68	16.3	33	7.9	7	1.7	131	31.4
	Total	65	15.6	199	47.7	125	30.0	28	6.7	417	100.0
24. I feel distressed when discussing my academic challenges with friends and family because it makes me uncomfortable.	Pet owner	40	9.6	92	22.1	112	26.9	42	10.1	286	68.6
	Non-pet owner	12	2.9	53	12.7	53	12.7	13	3.1	131	31.4
	Total	52	12.5	145	34.8	165	39.6	55	13.2	417	100.0
25. I often feel dissatisfied with the quality of support I receive in times of need.	Pet owner	51	12.2	125	30.0	80	19.2	30	7.2	286	68.6
	Non-pet owner	20	4.8	56	13.4	46	11.0	9	2.2	131	31.4
	Total	71	17.0	181	43.4	126	30.2	39	9.4	417	100.0
26. I find comfort in spending time with a pet, which relieves my stress.	Pet owner	2	0.5	11	2.6	64	15.3	209	50.1	286	68.6
	Non-pet owner	16	3.8	36	8.6	58	13.9	21	5.0	131	31.4
	Total	18	4.3	47	11.3	122	29.3	230	55.2	417	100.0
27. My engagement in leisure activities with a pet helps me cope with stress.	Pet owner	5	1.2	12	2.9	95	22.8	174	41.7	286	68.6
	Non-pet owner	11	2.6	36	8.6	59	14.1	25	6.0	131	31.4
	Total	16	3.8	48	11.5	154	36.9	199	47.7	417	100.0
28. A pet acting funny can amuse and entertain me.	Pet owner	2	0.5	9	2.2	48	11.5	227	54.4	286	68.6
	Non-pet owner	9	2.2	18	4.3	49	11.8	55	13.2	131	31.4
	Total	11	2.6	27	6.5	97	23.3	282	67.6	417	100.0
29. I find it hard to manage my budget and expenses associated with pet ownership.	Pet owner	37	8.9	111	26.6	108	25.9	30	7.2	286	68.6
	Non-pet owner	12	2.9	44	10.6	55	13.2	20	4.8	131	31.4
	Total	49	11.8	155	37.2	163	39.1	50	12.0	417	100.0
30. I feel like pets are messing with me while I work.	Pet owner	109	26.1	89	21.3	57	13.7	31	7.4	286	68.6
	Non-pet owner	31	7.4	58	13.9	32	7.7	10	2.4	131	31.4
	Total	140	33.6	147	35.3	89	21.3	41	9.8	417	100.0

Note: Statement 1–5: Personal Stressors; Statement 6–10: Coping Mechanisms; Statement 11–15: Impact of Physical Activity Levels; Statement 16–20: Impact on Well Being; Statement 21–25: Support System; Statement 26–30: Impact of Having a Pet on Stress.

**Table 3.** Perceived stress levels on different key stress areas among the pet owners and non-pet owners in Cebu, Philippines (*n* = 417).

Key stress area		Stress Levels							
		Low		Medium		High		Total	
		Freq	%	Freq	%	Freq	%	*n*	Relative %
Personal stressors	Pet owner	2	0.7	200	69.9	84	29.4	286	100.0
	Non-pet owner	1	0.8	78	59.5	52	39.7	131	100.0
Coping mechanisms	Pet owner	1	0.3	180	62.9	105	36.7	286	100.0
	Non-pet owner	1	0.8	90	68.7	40	30.5	131	100.0
Impacts of physical activities levels	Pet owner	55	19.2	197	68.9	34	11.9	286	100.0
	Non-pet owner	23	17.6	94	71.8	14	10.7	131	100.0
Impact on well-being	Pet owner	1	0.3	182	63.6	103	36.0	286	100.0
	Non-pet owner	1	0.8	75	57.3	55	42.0	131	100.0
Support system	Pet owner	6	2.1	227	79.4	53	18.5	286	100.0
	Non-pet owner	2	1.5	102	77.9	27	20.6	131	100.0
Impact of having a pet on stress	Pet owner	54	18.9	226	79.0	6	2.1	286	100.0
	Non-pet owner	3	2.3	106	80.9	22	16.8	131	100.0

A smaller fraction (2.4%) reported that pets served as a diversion from stressors, allowing a temporary escape from personal or work-related pressure. However, pet ownership also presents difficulties [37], with 8.9% of the respondents citing financial strain and time commitments [42]. These challenges indicate that pet ownership offers considerable advantages but demands responsibility and resource allocation, potentially increasing stress if not properly managed [14]. These results highlight the multifaceted relationship between pet ownership and mental well-being, in which emotional and physical benefits are balanced against practical challenges [40, 43]. Nevertheless, the overwhelmingly positive impact of pets on emotional support suggests that they play a vital role in stress management for many individuals.

This study highlights the complex relationship between pet ownership and stress levels. While pet owners reported lower levels of personal stress and higher engagement in coping mechanisms, pet care responsibilities posed challenges. The findings suggest that pet ownership may be a complementary stress-management strategy, particularly in enhancing emotional well-being and strengthening support systems. Further research is recommended to explore long-term effects and cultural influences on pet ownership and stress management in the Philippines.

This study used a non-probabilistic sampling method, and the findings may not be fully generalizable to the broader population. The sample’s limited diversity restricts the scope of conclusions. Its results should be interpreted with caution. The sample does not represent the Philippine population as a whole, as it focuses only on Cebu. Future research could benefit from more representative samples and exploring cultural factors that influence pet ownership and stress management in the Philippines.

## 4. Conclusions

Pet ownership is closely linked to emotional support and better stress management, especially among younger, single women. Pets offer companionship that helps reduce feelings of anxiety and loneliness while encouraging physical activity and social connection. The presence of pets may produce therapeutic emotional effects, easing loneliness and reducing anxiety by providing comfort, especially during times of stress. While pet ownership offers essential mental health benefits, it also comes with challenges such as financial costs and time demands, which can add to stress for some owners. Overall, having a pet appears to be a valuable tool for coping with daily pressures and enhancing well-being, though the experience varies by individual circumstances. These findings highlight the vital role pets play in many people’s lives as a source of comfort and support, while also reminding concerned individuals of the responsibilities that come with caring for them.

## Data Availability

The data presented in this study are available from the corresponding author upon reasonable request.

## List of abbreviations

SPSS, Statistical Package for the Social Sciences; Freq, frequency; *n*, number; *p*, statistical significance.

## References

[1] Shah SMA, Mohammad D, Qureshi MFH, Abbas MZ, Aleem S (2021). Prevalence, psychological responses and associated correlates of depression, anxiety and stress in a global population, during the coronavirus disease (COVID-19) pandemic. Community Ment Health J.

[2] Dominick W, Walenski-Geml A, Taku K (2020). Associations between pet ownership, posttraumatic growth, and stress symptoms in adolescents. Anthrozoos.

[3] Tee ML, Tee CA, Anlacan JP, Aligam KJ, Reyes PWC, Kuruchittham V (2020). Psychological impact of COVID-19 pandemic in the Philippines. J Affect Disord.

[4] Sharpley CF, Veronese N, López-Sánchez GF, Bitsika V, Demurtas J, Celotto S (2020). Pet ownership and symptoms of depression: A prospective study of older adults. J Affect Disord.

[5] Bhatia D, Bhatia A, Sarma D (2022). Impact of pet ownership and relationships on human psychological health and function. Gyan Manag J.

[6] Chaudhari A, Kartal T, Brill G, Amano KJ, Lagayan MG, Jorca D (2022). Dog ecology and demographics in several areas in the Philippines and its application to anti-rabies vaccination programs. Animals.

[7] Liu Y, Chang X, Yang S, Li Z, Wu Y (2024). “Pets make you spend more!” Impact of pet ownership on consumer purchase decisions. J Bus Res.

[8] McCune S, McCardle P, Griffin JA, Esposito L, Hurley KJ, Bures RM (2020). Editorial: Human-animal interaction (HAI) research: A decade of progress. Front Vet Sci.

[9] Aruta JJBR (2022). Socio-ecological determinants of distress in Filipino adults during COVID-19 crisis. Curr Psychol.

[10] Quing KAC, Baudin JSP, Maaliw RR (2024). A sequential explanatory study examining the buffering effects of human-animal interaction on stress and quality of life among work-from-home employees during the COVID-19 pandemic in the Philippines. COVID.

[11] Joseph N, Ashuthosh KC, D’souza AL, Shekar CB, Hariram S, Nayak AH (2019). Assessment of pet attachment and its relationship with stress and social support among residents in Mangalore city of south India. J Vet Behav.

[12] Friedmann E, Gee NR, Simonsick EM, Kitner-Triolo MH, Resnick B, Adesanya I (2023). Pet ownership and maintenance of cognitive function in community-residing older adults: Evidence from the Baltimore Longitudinal Study of Aging (BLSA). Sci Rep.

[13] McConnell AR, Brown CM, Shoda TM, Stayton LE, Martin CE (2011). Friends with benefits: On the positive consequences of pet ownership. J Pers Soc Psychol.

[14] Mueller MK, King EK, Callina KS, Dowling-Guyer S, McCobb E (2021). Demographic and contextual factors as moderators of the relationship between pet ownership and health. Health Psychol Behav Med.

[15] Le Roux MC, Wright S (2020). The relationship between pet attachment, life satisfaction, and perceived stress: Results from a South African online survey. Anthrozoös.

[16] Amano KJ, Kartal T (2017). Report on owned dog population survey in Cebu City, Philippines. WellBeing International.

[17] Chopik WJ, Oh J, Weidmann R, Weaver JR, Balzarini RN, Zoppolat G (2023). The perks of pet ownership? The effects of pet ownership on well-being during the COVID-19 pandemic. Perks of Pet Ownership.

[18] Barker SB, Knisely JS, McCain NL, Best AM (2005). Measuring stress and immune response in healthcare professionals following interaction with a therapy dog: A pilot study. Psychol Rep.

[19] Philippine Health Research Ethics Board (2017). National ethical guidelines for health and health-related research 2017.

[20] Lazarus RS, Folkman S

[21] DeVellis RF (2017).

[22] Salmon P (2001). Effects of physical exercise on anxiety, depression, and sensitivity to stress: A unifying theory. Clin Psychol Rev.

[23] Cohen S, Wills TA (1985). Stress, social support, and the buffering hypothesis. Psychol Bull.

[24] Walsh F (2009). Human-animal bonds I: the relational significance of companion animals. Fam Process.

[25] Astuti Y, Orhan BE, Setyawan H, Karacam A, Susanto N (2024). Exploring the connection between physical and mental health in women and dog ownership. Retos.

[26] Herzog H (2011). The impact of pets on human health and psychological well-being: Fact, fiction, or hypothesis?. Curr Dir Psychol Sci.

[27] Chen F (2020). Demographic factors and stress levels among pet owners: A cross-sectional study. J Demogr Res.

[28] Bolstad CJ, Porter B, Brown CJ, Kennedy RE, Nadorff MR (2021). The relation between pet ownership, anxiety, and depressive symptoms in late life: propensity score matched analyses. Anthrozoos.

[29] Clements H, Valentin S, Jenkins N, Rankin J, Gee NR, Snellgrove D (2021). Companion animal type and level of engagement matter: a mixed-methods study examining links between companion animal guardianship, loneliness and well-being during the COVID-19 pandemic. Animals.

[30] Allen J, Smith B, Johnson C (2022). Physiological and psychological benefits of pet ownership: A longitudinal study. J Health Psychol.

[31] Wong R, Co M (2023). 357 A case-control study to evaluate the impact of pet cat or dog ownership on medical students’ psychosocial well-being. Br J Surg.

[32] Brown D (2023). Implications of pet ownership studies for public health and policy development: A comprehensive review. J Public Health Policy.

[33] Caffrey-Casiano C (2016).

[34] Matijczak A, Tomlinson CA, Applebaum JW, Kogan LR, McDonald S (2024). Development and validation of the pet-related stress scale. Pets.

[35] Martins CF, Neill AR, Machová T (2023). Pet’s influence on humans’ daily physical activity and mental health: A meta-analysis. Front Public Health.

[36] Neill RD, Cunningham C, O’Doherty M, Smith L, Tully MA (2023). Pet ownership and physical activity in older adults: cross-sectional analyses from the NICOLA study. J Ageing Longev.

[37] Konstantinova A, Matasov V, Filyushkina A, Vasenev V (2021). Perceived benefits and costs of owning a pet in a megapolis: an ecosystem services perspective. Sustainability.

[38] Marí-Klose M, Marí-Klose P, Gallo P, Escapa S, Julià A (2025). Loneliness and pet ownership among dependent older adults in a Southern European urban context. Aging Ment Health.

[39] Downes MJ, Devitt C, Downes MT, More SJ (2017). Understanding the context for pet cat and dog feeding and exercising behaviour among pet owners in Ireland: A qualitative study. Ir Vet J.

[40] Wang H, Ng TK, Siu OL (2023). How does psychological capital lead to better well-being for students? The roles of family support and problem-focused coping. Curr Psychol.

[41] Kogan LR, Currin-McCulloch J, Bussolari C, Packman W, Erdman P (2021). The psychosocial influence of companion animals on positive and negative affect during the COVID-19 pandemic. Animals.

[42] Wan MM, Kelemen TK, Zhang Y, Matthews SH (2022). An island of sanity during COVID-19 pandemic: Does pet attachment support buffer employees’ stress due to job insecurity?. Psychol Rep.

[43] Barklam EB, Felisberti FM (2023). Pet ownership and well-being during the COVID-19 pandemic: The importance of resilience and attachment to pets. Anthrozoos.

